# Synoptic assessment of coastal total alkalinity through community science

**DOI:** 10.1088/1748-9326/abcb39

**Published:** 2021-01-21

**Authors:** J E Rheuban, P R Gassett, D C McCorkle, C W Hunt, M Liebman, C Bastidas, K O’Brien-Clayton, A R Pimenta, E Silva, P Vlahos, R J Woosley, J Ries, C M Liberti, J Grear, J Salisbury, D C Brady, K Guay, M LaVigne, A L Strong, E Stancioff, E Turner

**Affiliations:** 1 Woods Hole Oceanographic Institution, Department of Marine Chemistry and Geochemistry, Woods Hole, MA 02543, United States of America; 2 Woods Hole Oceanographic Institution, Woods Hole Sea Grant, Woods Hole, MA 02543, United States of America; 3 University of Maine, Orono, ME 04469, United States of America; 4 Maine Sea Grant, Orono, ME 04469, United States of America; 5 Woods Hole Oceanographic Institution, Department of Geology and Geophysics, Woods Hole, MA 02543, United States of America; 6 University of New Hampshire, Durham, NH 03824, United States of America; 7 US Environmental Protection Agency Region 1, Boston, MA 02109, United States of America; 8 MIT Sea Grant, Cambridge, MA 02139, United States of America; 9 Connecticut Department of Energy and Environmental Protection, Hartford, CT 06106, United States of America; 10 US Environmental Protection Agency, Atlantic Coastal Environmental Sciences Division, Narragansett, RI 02882, United States of America; 11 Northeastern Regional Association of Coastal Ocean Observing Systems (NERACOOS), Portsmouth, NH 03801, United States of America; 12 University of Connecticut, Storrs, CT 06269, United States of America; 13 Massachusetts Institute of Technology, Center for Global Change Science, Cambridge, MA 02139, United States of America; 14 Northeastern University, Marine Science Center, Department of Marine & Environmental Science, Nahant, MA 01908, United States of America; 15 Bowdoin College, Department of Earth and Oceanographic Science, Brunswick, ME 04011, United States of America; 16 Hamilton College, Environmental Studies Program, Clinton, NY 13323, United States of America; 17 University of Maine Cooperative Extension Office, Waldoboro, ME 04572, United States of America; 18 National Oceanic and Atmospheric Administration, National Centers for Coastal Ocean Science, Silver Spring, MD 20910, United States of America, Retired; 19 Equally contributing first author

**Keywords:** ocean acidification, coastal acidification, total alkalinity, community science, citizen science, NECAN

## Abstract

Comprehensive sampling of the carbonate system in estuaries and coastal waters can be difficult and expensive because of the complex and heterogeneous nature of near-shore environments. We show that sample collection by community science programs is a viable strategy for expanding estuarine carbonate system monitoring and prioritizing regions for more targeted assessment. ‘Shell Day’ was a single-day regional water monitoring event coordinating coastal carbonate chemistry observations by 59 community science programs and seven research institutions in the northeastern United States, in which 410 total alkalinity (TA) samples from 86 stations were collected. Field replicates collected at both low and high tides had a mean standard deviation between replicates of 3.6 ± 0.3 *μ*mol kg^−1^ (*σ*_mean_ ± SE, *n* = 145) or 0.20 ± 0.02%. This level of precision demonstrates that with adequate protocols for sample collection, handling, storage, and analysis, community science programs are able to collect TA samples leading to high-quality analyses and data. Despite correlations between salinity, temperature, and TA observed at multiple spatial scales, empirical predictions of TA had relatively high root mean square error >48 *μ*mol kg^−1^. Additionally, ten stations displayed tidal variability in TA that was not likely driven by low TA freshwater inputs. As such, TA cannot be predicted accurately from salinity using a single relationship across the northeastern US region, though predictions may be viable at more localized scales where consistent freshwater and seawater endmembers can be defined. There was a high degree of geographic heterogeneity in both mean and tidal variability in TA, and this single-day snapshot sampling identified three patterns driving variation in TA, with certain locations exhibiting increased risk of acidification. The success of Shell Day implies that similar community science based events could be conducted in other regions to not only expand understanding of the coastal carbonate system, but also provide a way to inventory monitoring assets, build partnerships with stakeholders, and expand education and outreach to a broader constituency.

## 1. Introduction

Ocean and coastal acidification (OCA) has emerged during the last decade as a topic of serious concern, because of its impacts on marine organisms and coastal economies [1, 2]. There is a strong scientific consensus about the drivers and projections of ocean acidification in the open ocean, but the dynamics of acidification in coastal ecosystems are less clear. In addition to absorption of carbon dioxide, the coastal carbonate system is driven by a number of factors including freshwater discharge, stratification, water residence time, eutrophication, biogeochemical processes, and upwelling [3–11]. Natural biogeochemical cycling, which can be strengthened by eutrophication, combined with site-specific differences in tidal flushing and water residence time, leads to large spatio-temporal variations in seawater carbonate system parameters [11–17].

Many drivers of OCA are strongly localized and are likely determined by characteristics specific to both watersheds and estuaries, rendering regional generalizations of OCA conditions difficult. As a result, the OCA research community has identified the need for additional monitoring to better understand the drivers of the coastal carbonate system and quantify localized risk of future OCA [18–21]. Monitoring conditions across multiple spatial and temporal scales is important for developing models that inform management and for identifying and prioritizing opportunities for mitigation and adaptation (e.g. [22–24]; see also state OCA Action Plans).

OCA risk assessments for coastal regions involve comprehensive analyses of current and potential future biogeochemical conditions and prediction of the ecological consequences of OCA, combined with knowledge of societal impacts within specific estuaries (e.g. [2, 25, 26]). Marine calcifiers are particularly at risk from OCA, and mollusks at most life stages are sensitive to reduced carbonate mineral saturation state (Ω) and pH (e.g. [27, 28]). Previous studies have consistently shown negative effects of OCA on critical shellfish life history stages, including fertilization, shell formation, and larval development (e.g. [18], and references therein, [29–31]). Reduced shell strength and growth, increased mortality, and altered behavior have been shown for juveniles and adults of some species, although responses in laboratory experiments have been variable, and both species and sub-population specific [18, 32–38]. Because of the strong potential sensitivity of mollusks to OCA and limited mobility within a coastal estuary, areas with significant wild shellfish populations and aquaculture operations are candidate locations for enhanced monitoring and determination of localized drivers of the carbonate system.

Total alkalinity (TA), a measure of the ability of a solution to resist a change in pH, is one of four parameters that describes the seawater carbonate system. Sample collection is straightforward due to the lack of sensitivity of TA to gas exchange, and sample storage over short periods of time does not require inhibition of biological activity with poisoning agents such as mercuric chloride [39, 40]. In the absence of biological processes, TA is also conservative with salinity, and relationships between salinity and TA have been used in the monitoring of coastal acidification [41]. TA can be a useful indicator of marine ecosystems’ vulnerability to acidification pressure from various CO_2_ sources; however, TA is not typically monitored by community science organizations because of financial and analytical barriers. The widespread adoption of community science for water quality monitoring has overcome these hurdles for other parameters, e.g. temperature, salinity, dissolved oxygen, and nutrients [42–45]. In addition, community science has proven to be important for public outreach, engagement, education, and adoption of practices that expand and promote environmental stewardship. Many monitoring organizations have also been vocal advocates of the development and implementation of management solutions to improve coastal water quality such as garnering support for nutrient pollution regulation, upgrades to wastewater treatment facilities, and expansion of sewer networks. Expanding site-specific monitoring programs to include observations of coastal carbonate chemistry may be a capacity-building step toward public education and the implementation of local management actions to reduce the drivers of acidification [22, 24, 46, 47].

We carried out ‘Shell Day’ on 22 August 2019, as a synoptic water monitoring event coordinating coastal TA observations among community science programs and research institutions from Long Island Sound (LIS) to Downeast Maine. To our knowledge, this study represents the first large-scale set of synchronous measurements of salinity and TA along the northeastern United States coast. The sampling was motivated by two years of outreach and capacity-building activities by the Northeast Coastal Acidification Network aimed at training community-based water monitoring programs in methods to measure carbonate chemistry parameters [40, 47], an effort to be detailed in a companion manuscript, Gassett *et al* [48]. Shell Day had three major goals: (a) to evaluate the efficacy of community science for TA monitoring; (b) to assess geographic heterogeneity in mean and tidal variability in TA; and (c) to determine if a regional relationship between salinity, temperature, and TA could be used to estimate TA. This manuscript describes the successes, shortcomings, and uncertainties in achieving these goals.

## 2. Methods

### 2.1. Site selection and sampling design

Minimum requirements for participation in Shell Day were the capacity to measure water temperature and salinity; some organizations used thermometers and refractometers, and others used multiparameter datasondes or handheld units. Fifty-nine water monitoring organizations participated, collecting samples from 86 stations. Sampling stations were chosen by individual monitoring organizations, with suggested criteria for choosing locations including: stations with long sampling records, proximity to wild shellfish populations, shellfish aquaculture operations or hatcheries, stations with relatively easy access to facilitate repetitive sampling, and/or stations with large variability in salinity.

An Environmental Protection Agency (EPA) approved quality assurance project plan was developed along with a datasheet, sampling protocol, training video, and a webinar tutorial to instruct community scientists on a standardized sampling protocol (all available at necan.org/shellday). Surface water samples were collected in pre-cleaned and labeled borosilicate glass or HDPE bottles provided to participating organizations. Samples were collected at low, mid, and high tides at each station to assess the tidal variation of TA. Samples were collected directly from the water body or using common sampling devices such as buckets, Van Dorn samplers, or Niskin bottles. Temperature, salinity, and other water characteristics were measured either directly in the water body at the targeted depth of sample collection (handheld units and multiparameter datasondes) or from the water collected in a container (refractometers). To assess the consistency of the sampling protocol, sample handling, and sample storage, field duplicates were collected at both low and high tides. Water samples were placed on ice and stored in the dark upon collection and either returned to the lab on the day of collection or stored overnight on ice until samples could be returned to the nearest laboratory. Upon delivery to a laboratory, water samples were either analyzed immediately or fixed by laboratory staff with saturated mercuric chloride to inhibit biological activity and analyzed over several weeks. Participants were also asked to provide metadata such as location (upper/mid/lower estuary), proximity to wild shellfish populations or aquaculture operations (yes/no/unknown), and other information such as salinity instrument type and date of last calibration. In order to ensure safety of volunteers collecting water samples, participants were notified during the pre-sampling training webinar about inclement weather plans, and no volunteers handled hazardous laboratory materials.

Spatial data layers from the Northeast Ocean Data Portal (NODP) on commercial aquaculture operations [49] and shellfish habitat suitability [50] were used to corroborate participant responses and identify other sampling stations located within 1 km of wild shellfish populations and aquaculture operations.

### 2.2. Sample processing

Seven laboratories analyzed samples for TA via automated open-cell Gran titration (Method 1: [51]) or modified single-point titration (Method 2: [52]) (table 1). Each laboratory used certified reference material (CRM) from Dr A G Dickson’s laboratory at the Scripps Institute of Oceanography to standardize measurements. Although an inter-laboratory comparison would increase the confidence in and comparability of our results, such comparison was beyond the scope of this study. TA data were quality controlled by each laboratory based on instrument performance, laboratory replicates, and analyses of CRM. Data were excluded from analyses if the standard deviation between field duplicates was greater than 1% of the mean. Reported salinity was converted from practical salinity to absolute salinity using the Gibbs Seawater Matlab toolbox [53]. A subset of 11 samples had salinities verified on a benchtop salinometer (Guildline Portasal).

### 2.3. Data analysis

**Figure 1.**
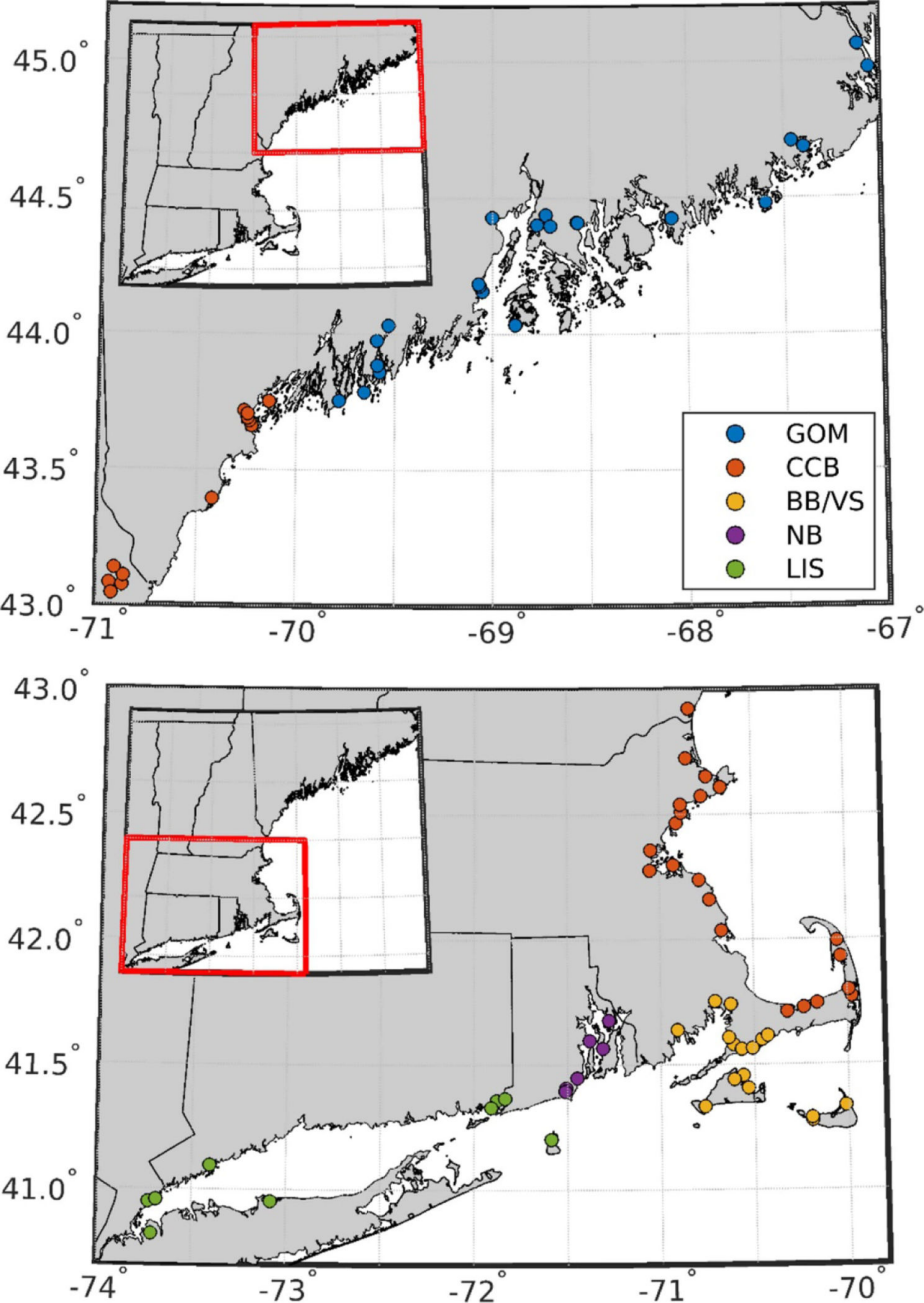
Sampling locations. Sampling stations are colored by geographic groupings corresponding to regions in tables 2 and 3, and figure 3. Groupings are the northern Gulf of Maine (GOM, blue), central Gulf of Maine/Cape Cod Bay (orange, CCB), Buzzards Bay/Vineyard Sound (BB/VS, yellow), Narragansett Bay (NB, purple), and Long Island Sound (LIS, green).

Empirical relationships between physical variables (temperature, salinity, latitude) and TA were evaluated for both the entire dataset and groupings of stations by subregion (figure 1) using simple linear regression (salinity only) and multiple linear regression (MLR) using equations with similar form as Juranek *et al* [54] and Alin *et al* [3]:

(1) $${\text{TA}}_{\text{Northeast}\;}=A_{1}+A_{2}\left(S-S_{\text{r}}\right)+A_{3}\left(T-T_{\text{r}}\right)\;+A_{4}(\;\text{Lat}\;)+A_{5}\left(S-S_{\text{r}}\right)\left(T-T_{\text{r}}\right)\;+A_{6}\left(S-S_{\text{r}}\right)\times \;\text{Lat}\;+A_{7}\left(T-T_{r}\right)\times \;\text{Lat}.$$

(2) $${\text{TA}}_{\text{i}}=A_{1,\text{i}}+A_{2,\text{i}}\left(S_{\text{i}}-S_{\text{r},\text{i}}\right)+A_{3,\text{i}}\left(T_{\text{i}}-T_{\text{r},\text{i}}\right)\;+A_{4,\text{i}}\left(S_{\text{i}}-S_{\text{r},\text{i}}\right)\left(T_{\text{i}}-T_{\text{r},\text{i}}\right)$$

where TA is total alkalinity, *S* is salinity, *T* is temperature, Lat is latitude, the r-subscript indicates a reference temperature and salinity, defined as the mean temperature or salinity for the dataset analyzed and the subscript i indicates subregions. Subregion delineation was informed by Gledhill *et al* [18], with station groupings including LIS, Narragansett Bay (NB), Buzzards Bay/Vineyard Sound (BB/VS), Cape Cod Bay/Central Gulf of Maine (CCB), and northern Gulf of Maine (GOM) (see figure 1). Latitude was only included as a predictor variable when evaluating the entire dataset (equation (1)).

## 3. Results

A total of 410 samples were collected. Field duplicates were collected at low and high tides at most stations, leading to 264 unique samples. Eight sets of field duplicates with a%-standard-deviation from the mean of more than 1% were excluded from this analysis. There was good agreement between the remaining pairs with a mean standard deviation between duplicates of 3.6 ± 0.3 *μ*mol kg^−1^ (±SE, *n* = 145) or 0.20 ± 0.02%. The TA of 122 of 145 sets of duplicates (84.1%) differed by less than 10 *μ*mol kg^−1^. Laboratory verification of a subset of salinity measurements (*n* = 11) showed average differences between field and lab salinity (±SD) of 1.9 ± 2.1, and salinometer measurements were used in place of field observations where available. Additionally, ten field salinity values were higher than typically observed in the coastal New England region (>34) and were excluded from the interpretation.

**Figure 2.**
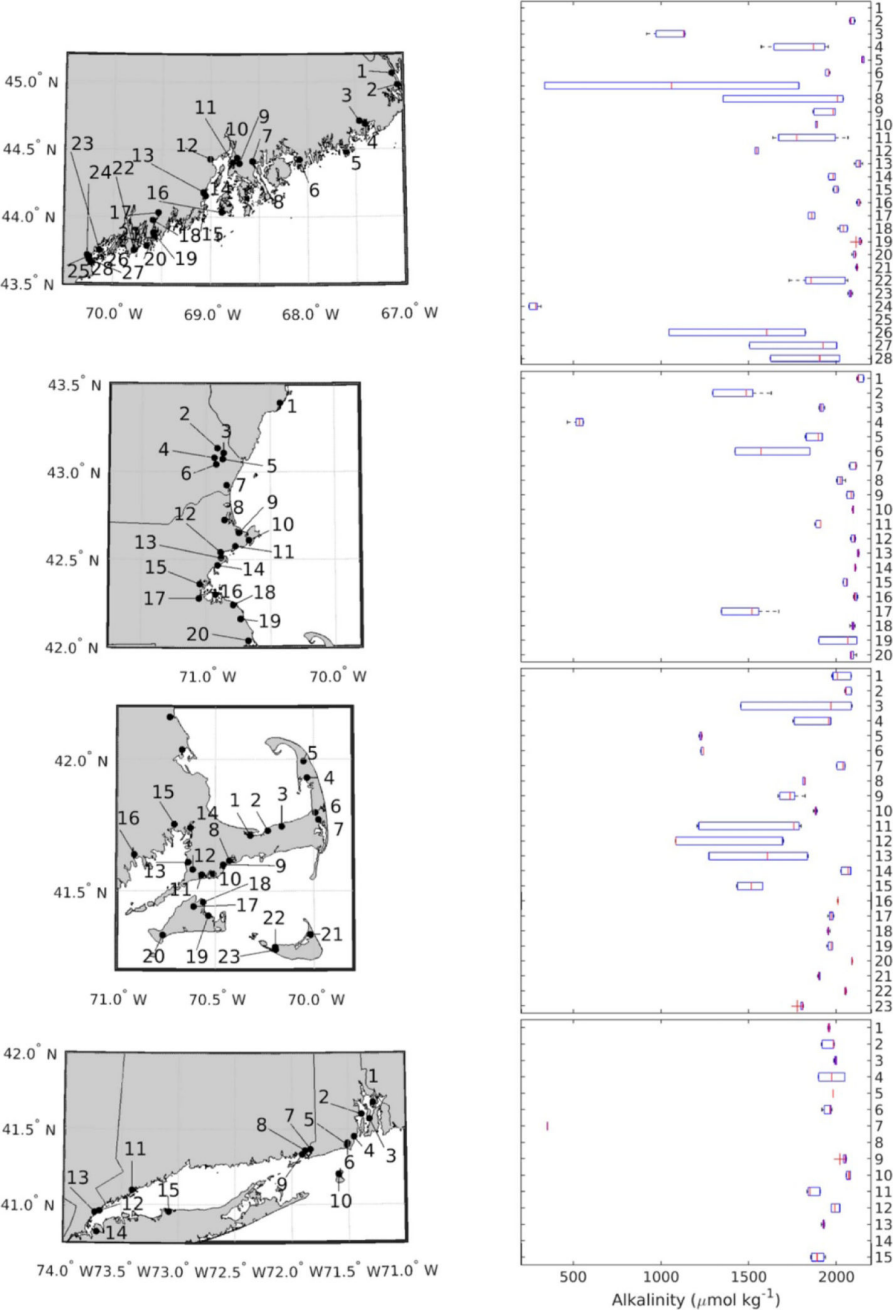
Left panels show subsets of locations of sampling stations moving from north to south down the Northeast US coast: northeastern Maine (top), southwestern Maine, New Hampshire, northern Massachusetts (2nd), Cape Cod, Martha’s Vineyard, and Nantucket, Massachusetts (3rd), Rhode Island, Connecticut, and New York (bottom). Right panels show distribution of station alkalinity measurements. Boxplots are generated using all data from each station, representing up to six samples collected. Station numbers for each boxplot correspond to numbers in the map panels on the left. Red lines indicate the station median, the box the interquartile range, and whiskers correspond to ±2.7*σ*.

**Figure 3.**
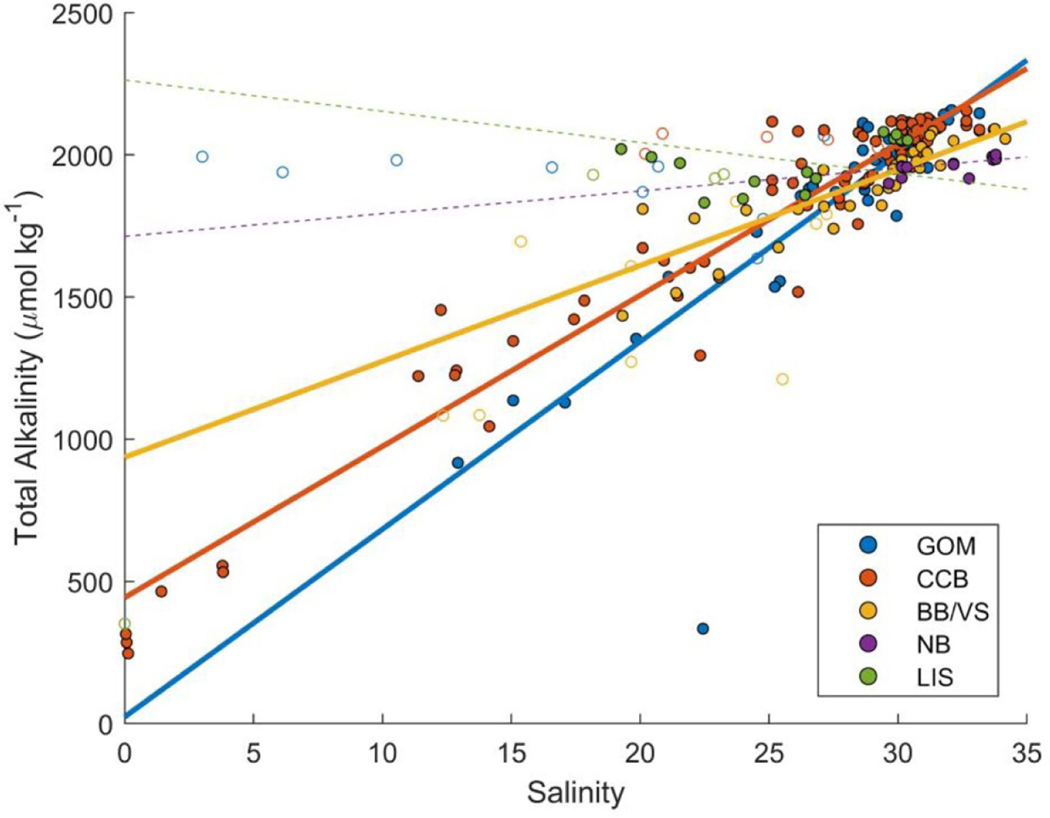
Salinity-total alkalinity (TA) relationships. Each station is represented by at most three points, showing the TA and salinity values for low, mid, and high tide, as available. Open circles are from the ten stations where tidal variability in salinity and TA was unexpected. Data points are colored by regional grouping shown in figure 1. Lines are calculated from regression analyses using the mean temperature for each region. Dashed lines for LIS and NB are included for completeness, but the slopes with respect to salinity were not statistically significant (*p* > 0.05, table S1).

There was a high degree of geographic variation in TA (table 2), and also in station mean and distribution over a tidal cycle (figure 2). Simple regression analysis indicated a correlation between salinity and TA across the entire dataset (*r*^2^ = 0.668, *p* < 0.0001, not shown) that was improved (*r*^2^ = 0.820, *p* < 0.0001, table S1 and figure S1 (which are available online at stacks.iop.org/ERL/16/024009/mmedia)) by excluding data from stations where high tidal variability in either salinity or TA was not accompanied by variability in the other parameter (figure 3, open circles, see paragraph below). The best correlations, with both the highest *r*^2^ and lowest root mean squared error (RMSE), were achieved by incorporating temperature (for all data combined and by subregion) and latitude (for all data combined) as predictor variables via MLR (tables 3 and S2, figure 3 filled circles only). Despite a relatively high *r*^2^ for fits combining all data, RMSE was large (121.1 *μ*mol kg^−1^). Analyzing the data in groupings by subregion improved the prediction for some regions and worsened the prediction for others (figure 3, tables 3, S1 and S2).

**Figure 4.**
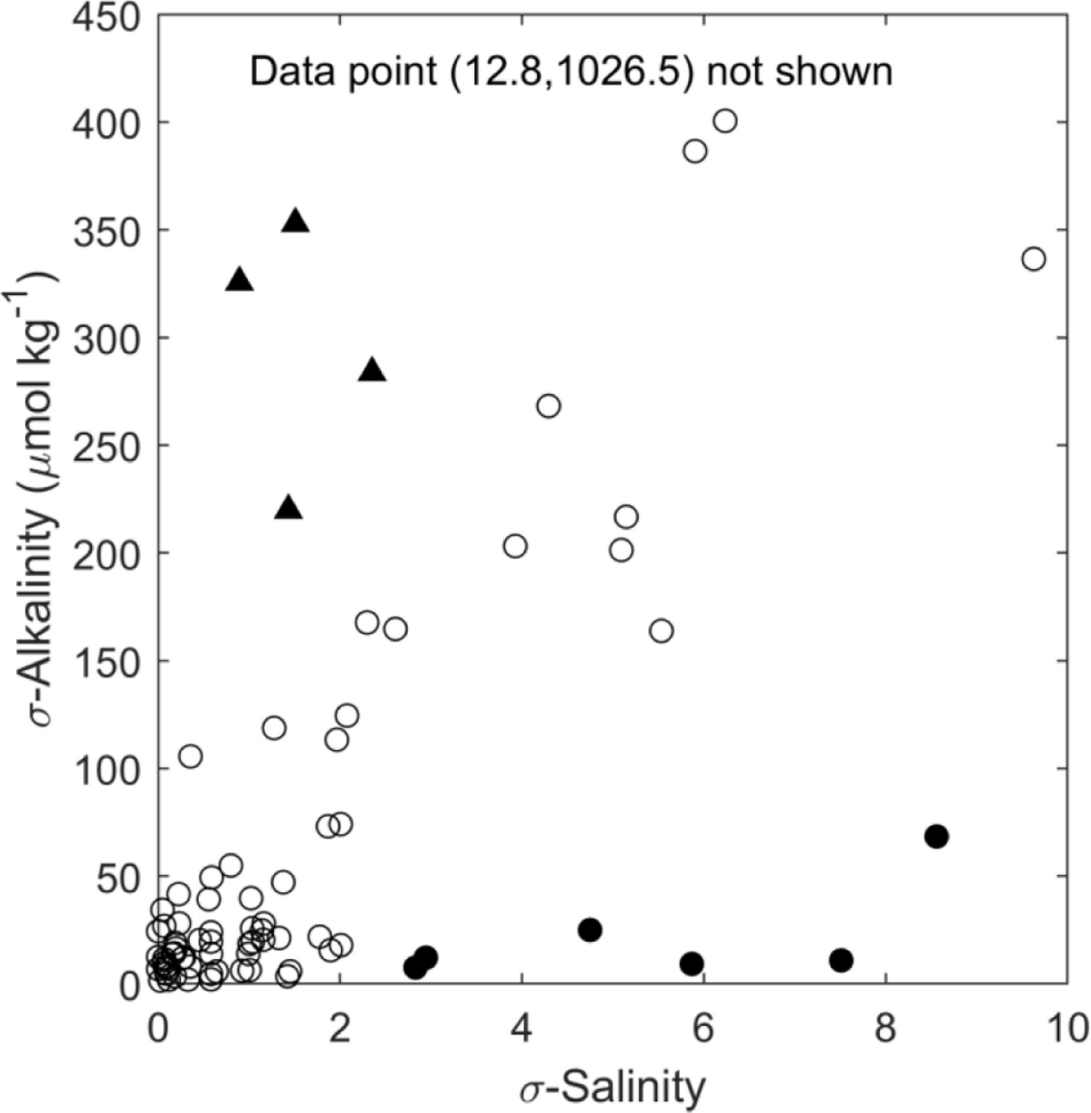
Standard deviation in total alkalinity vs standard deviation in salinity over a tidal cycle from each sampling station. Open circles are stations with low or proportional variation in salinity and alkalinity (group 1), closed triangles have large variation in alkalinity but small variation in salinity (group 2), and closed circles have large variation in salinity but small variation in alkalinity (group 3).

Station-level standard deviation was used to assess tidal variations in TA and salinity (figure 4).Three patterns of variation were identified: stations with (a) low or proportional variation in both TA and salinity; (b) low variation in salinity but high variation in TA (*σ*_TA_ > 200*μ*mol kg^−1^, *σ*_sal_ < 2.5); (c) high variation in salinity but low variation in TA (*σ*_sal_ > 2.5, *σ*_TA_ < 100 *μ*mol kg^−1^). The majority of sampling stations fell into the first (*n* = 71) category, four into the second category, and six into the third category. Five stations did not have enough samples to evaluate variability over a tidal cycle.

Participants identified 47 sampling locations that they believed were in close proximity to shellfish aquaculture or wild shellfish populations. Spatial data layers from the NODP identified 18 and 58 stations within 1 km of shellfish aquaculture operations or suitable shellfish habitat, respectively. Some stations overlapped these three data sources, and combining data sources yielded 72 stations monitored on Shell Day that were likely near shellfish populations. Five stations in close proximity to shellfish had mean TA that was lower than the 20th percentile in the dataset (TA < 1750.7 *μ*mol kg^−1^), six stations had tidal variability in TA greater than the 80th percentile (*σ*_TA_ > 120.4 *μ*mol kg^−1^), and six stations had both low mean and high tidal variability.

## 4. Discussion

### 4.1. Efficacy of sampling design

To the authors’ best knowledge, Shell Day was the most geographically extensive, single-day effort to sample and analyze carbonate system parameters of seawater in coastal New England (figure 1, table 2), and the first to evaluate a community science strategy for discrete carbonate system monitoring. Individual sampling programs have carried out more comprehensive monitoring of single embayments and estuaries (e.g. [11, 17, 55, 56]), but the single-day sampling over a tidal cycle provides a unique snapshot of variability in TA and salinity across both time and space (figure 2). Long term and high-precision observations may be required to discern location-specific drivers of OCA, but synoptic approaches such as Shell Day can help prioritize locations for more targeted assessments (see section 4.4).

The good agreement between field replicates indicates that the community-based sample collection and handling protocols generally yielded high-quality TA measurements (figure S1). The success of Shell Day suggests that community science organizations with capacity for additional water sample collection could collaborate with laboratories to add TA to sampling programs to improve understanding of OCA, while increased community science participation can serve to facilitate long-term observations. Such combined science and outreach efforts may also help communities and managers better understand the complex dynamics of OCA and increase public engagement in addressing this environmental challenge.

### 4.2. Empirical relationships between salinity, temperature and total alkalinity

The entire salinity-TA data set displays high variability (figure 3) and suggests that dilution with low TA freshwater exerts a strong control on coastal TA across the Northeast (figure 3, filled symbols). However, ten Shell Day sampling stations exhibited strong deviations from this overall trend (figure 3, open symbols), illustrating the potential importance of other localized processes. For instance, several stations exhibited TA of ca. 2000 *μ*mol kg^−1^ and reported salinity that approached zero, implying differences in freshwater endmembers at the watershed scale (see section 4.3). Additionally, the geographic subsets of the data identified in figure 1 show distinct salinity-TA relationships, with differences in both the salinity-TA slope and the zero-salinity intercept. Many environmental and water quality factors may vary at the subregion and watershed scale, such as underlying gradients in both coastal and freshwater endmembers spanning this large region (e.g. figure S4, [18]) that could contribute to these differences in salinity-TA regressions (figure 3).

One project goal was to determine if a region-wide empirical relationship could be used to accurately estimate TA from salinity and temperature, two parameters typically monitored by community science programs. Although strong regional relationships between salinity, temperature, and TA have been observed in other studies (e.g. [3, 54, 57–59]), the Shell Day data suggest there is not likely to be a single Northeast-wide relationship that can accurately predict TA in coastal waters. For the entire region, the best empirical relationship still had a high RMSE (121.1 *μ*mol kg^−1^), which contrasts with the tight salinity-TA relationships observed in the more open ocean environments of the Northwest Atlantic continental shelf [16, 60]. Even by subregion, RMSE was still high (>48 *μ*mol kg^−1^) in all statistically significant empirical fits (table 3). This error is 10–50 times greater than the laboratory measurement uncertainty, which is typically on the order of 1–4 *μ*mol kg^−1^.

Ultimately, the goal of empirical fits to predict TA should be to achieve a low RMSE so as to limit additional uncertainty in carbonate system calculations. For example, Alin *et al* [3] and Juranek *et al* [54] predicted TA with an overall RMSE of 6.4 and 4.8 *μ*mol kg^−1^, in the Southern California Coastal Current and Northeast Pacific regions, respectively. In the nearshore and estuarine environments of Washington state, Fassbender *et al* [59] predicted TA from salinity with an error of 17 *μ*mol kg^−1^, which they estimated was appropriate for ‘weather’ quality calculations of Ω and pH, but not ‘climate’ quality calculations [61], where a prediction error on TA needed to be <10 *μ*mol kg^−1^. A similar error propagation analysis to determine maximum error acceptable for TA predictions is unfortunately not possible with this dataset owing to the lack of additional carbonate system measurements. Thus, given this high uncertainty, salinity and temperature alone, at least as measured in the Shell Day dataset, are not sufficient to estimate TA across the entire Northeast United States region. However, empirical relationships to predict TA may be possible using more localized datasets. This is supported by the reduced error when predicting TA from salinity and temperature for some of the subregions, reinforcing the need to understand drivers of the carbonate system at the watershed scale (tables 2 and S1).

Several factors could drive the high predictive error when estimating TA from proxies in the coastal environment of the Northeast. Processes that influence TA such as sulfate reduction, denitrification, calcification, or calcium carbonate dissolution could be responsible for some of the variation in the salinity-TA relationship, but we lacked the data to evaluate the contribution of these processes. Challenges with the collection of high-quality salinity data may also have led to increased variability in the salinity-TA relationship. For instance, imprecise calibration of handheld salinity meters or lower precision and accuracy of refractometers (used at 23 of the sampling locations) may have contributed to this poor correlation. Furthermore, if the water column was stratified at the time of sample collection, small differences between the depth of the salinity measurement and the depth that the water was sampled for TA analysis could lead to decoupling of salinity from TA. Empirical fits between temperature, salinity, and TA using only laboratory salinometer measurements on the water collected for TA analysis in the GOM region, rather than field observations, showed much smaller RMSE (30.1 vs 177.1 *μ*mol kg^−1^, respectively) and higher *r*^2^ values (0.993 vs 0.772, respectively) (tables S1 and 3) than the overall GOM region, although this dataset was also much smaller. The reduced error implies that more accurate measurements of salinity may improve the predictive capacity of a subregional empirical relationship, although more data would be needed to fully evaluate this hypothesis.

### 4.3. Carbonate system variability

Station-level standard deviation values for salinity and TA provide insight into factors that influence tidal variability in TA during the time of sampling (figure 4). For instance, stations that displayed little or proportional variability in both TA and salinity (Group 1, figure 4) likely illustrate conservative mixing with low-TA freshwater as the dominant driver of TA variability over a tidal cycle. Most of the observations fall into this category, which reflects the strong impact of freshwater inputs on TA concentration (see also section 4.2). Stations with low variability in both TA and salinity may reflect either coastal or riverine endmembers, but given the limited nature of this dataset (three samples per station) and relatively high uncertainty in reported salinity (1.9 ± 2.1), it is not possible to distinguish natural variability in salinity from measurement uncertainty for observations where *σ*_salinity_ was less than approximately 2 units.

Stations with large changes in TA but low salinity variation (Group 2, figure 4) could be influenced by alkalinity production from sediments [62–65]. For example, at a single sampling station, Wang *et al* [64] observed a nearly 200 *μ*mol kg^−1^ increase in TA from high to low tide during summer, which was attributed to anoxic or suboxic processes occurring in marsh sediments such as sulfate reduction or denitrification that led to significant alkalinity export during ebb tide. Production of dissolved organic carbon can also lead to increased contributions of organic alkalinity that could cause tidal variations in TA without changes in freshwater inputs [66]. Organic acids have been estimated to modify coastal TA by up to 100 *μ*mol kg^−1^ [64, 66, 67], potentially representing 20%–50% of the signal observed at these four sampling stations.

The six stations with large variation in salinity but little change in TA over a tidal cycle (Group 3) may reflect high alkalinity freshwater contributions. Compilations of river alkalinity measurements collected over the past several decades indicate that most observations of freshwater TA in the New England region are relatively low (200–1000 *μ*mol kg^−1^, [17, 56, 68], figure S4) in comparison to expected seawater values (>2000 *μ*mol kg^−1^), but a number of rivers that discharge into coastal Maine, New Hampshire, northern Massachusetts, and LIS have much higher TA (>1000 *μ*mol kg^−1^, [55, 68], figure S4). However, more data along with repeat sampling over multiple tidal cycles would be needed to better understand these anomalous relationships between TA and salinity.

### 4.4. Using distributed monitoring for targeted assessments

**Figure 5.**
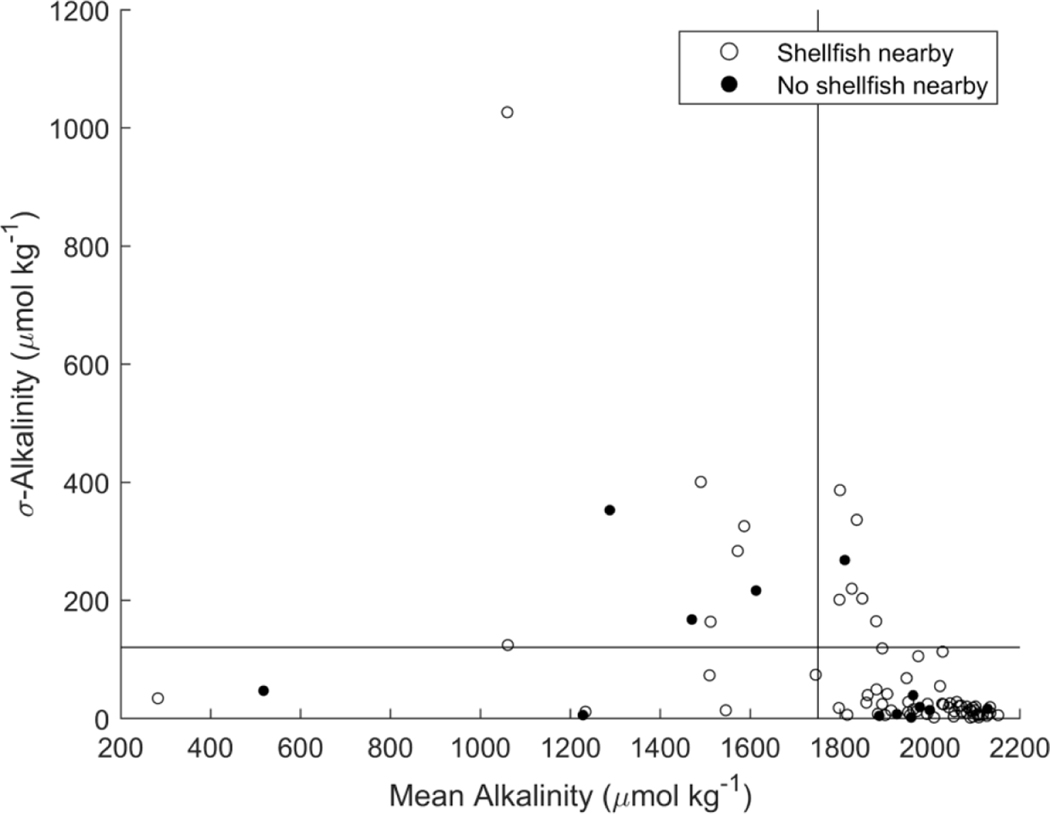
Station mean and standard deviation in total alkalinity. Open circles are locations identified as near shellfish aquaculture, wild populations, or suitable shellfish habitat. Closed circles indicate stations not adjacent to aquaculture or wild shellfish habitats. Vertical and horizontal lines indicate the extreme low and high values (20th and 80th percentiles for mean and standard deviation, respectively) in the distribution of the Shell Day data.

Assessment of the vulnerability of communities, economies, and ecosystems to OCA requires detailed syntheses of social and biogeochemical conditions (e.g. [26, 69]). No single sampling program could provide those syntheses, but efforts like Shell Day may help identify locations for in-depth evaluation of vulnerability to OCA. Although explicit biological thresholds for mean or variability in TA for shellfish are not known, extreme values within the distribution of the Shell Day dataset may be used to suggest locations for further study. For example, stations in close proximity to shellfish that also had low mean TA, high tidal variability in TA, or both, are likely to experience higher levels of coastal acidification stress, or be at risk for future acidification due to low buffering capacity (figure 5, [18]). In addition, DIC tends to be higher than TA in rivers and groundwater in New England [17, 56, 70] and carbonate system buffering diminishes as DIC increases relative to TA [64, 71–73]. Thus, regions of low TA, especially if caused by mixing with high DIC, low TA freshwater, are likely to have a higher sensitivity to future increases in CO_2_ from either atmospheric or local biological sources. Highly variable environments have also been proposed as locations that promote adaptation and/or evolution of resilience to acidification stress ([74] and references therein), and the distributed, single-day monitoring approach may identify potentially resilient populations of shellfish.

### 4.5. Recommendations for community-based sampling and measurements of seawater parameters

These results suggest two important practical considerations for future studies. First, the development of empirical relationships between salinity and TA relies on high-quality measurements of both salinity and TA, but during Shell Day, salinity was measured less accurately than anticipated. Refractometer-based salinity measurements often differed from laboratory measurements by several units, and even sensor-based salinity measurements sometimes yielded implausible values. These problems emphasize the importance of careful, well-documented calibration and verification procedures. In addition, in a strongly stratified water column, salinity measured in the water column using handheld instruments may differ from the actual salinity of the discrete water sample collected for TA analysis. At a minimum, salinity and TA should be measured at precisely the same water depth or, ideally, salinity should be measured on a subsample of the water sample used for TA measurements.

More broadly, Shell Day responds to the call to expand coastal monitoring, build partnerships that utilize existing monitoring efforts to observe coastal carbonate chemistry, and increase education and outreach on behalf of OCA as an indicator of climate change and water quality [46]. A natural expansion of this approach would be the addition of a second carbonate system parameter. The cost and calibration of equipment poses challenges for *in situ* measurements of pH and pCO_2_, while bottle sampling for dissolved inorganic carbon, pH, or pCO_2_ has significant challenges related to the collection, handling, and preservation of samples that are sensitive to gas exchange. Adjustments would need to be made to the sampling protocol, such as providing sampling devices designed to minimize gas exchange, using gas-impermeable borosilicate glass bottles, and more rapid preservation of samples immediately after collection. These approaches may not be appropriate for community science because sample preservation typically involves using a concentrated solution of mercuric chloride, a hazardous substance.

Community science driven seawater monitoring can serve many purposes. This project was designed to pilot a community science based approach to characterizing single-day variations in TA across a large geographic range. A targeted sampling design prioritizing specific ecosystems, communities of interest, and/or drivers of coastal carbonate chemistry could be developed in collaboration with academic researchers to address specific questions or enhance evaluation of regional differences in vulnerability to acidification. For example, the EPA’s National Coastal Condition Assessment added TA to its suite of standard measured parameters beginning in 2020. Future efforts could also target sites with shellfish aquaculture or large populations of wild shellfish, or be timed to address specific processes, such as the spring freshet, peak respiration, major storm events, or seasonal patterns of eutrophication.

## 5. Conclusions

The success of the Shell Day sampling effort illustrates the potential of community science to contribute to carbonate system monitoring. These results reveal ways to improve sampling methodology and show the value of TA as a potential tool for OCA studies. This project highlighted opportunities for laboratories and research facilities to collaborate with coastal monitoring programs and community science organizations, developed resources that could be used to support future events at other locations (e.g. Quality Assurance Project Plan, data sheets, sampling protocols, educational documents and videos), and identified areas of expansion such as procedures to collect samples for other carbonate system parameters. Community science efforts can provide a way for state and local governments to inventory monitoring assets, establish collaborations among laboratories to build capacity for seawater monitoring, and engage constituencies in education and outreach programs that increase public understanding of ocean and coastal acidification.

## Supplementary Material

Sup 1

## Figures and Tables

**Table 1.** List of laboratory facilities and instruments. Samples were analyzed for TA via automated open-cell gran titration (1) or modified single-point titration (2).

Partnering laboratory	Titrator brand	Method

Bowdoin College	Metrohm 905 Titrando	1
EPA Atlantic Coastal Environmental Sciences Division	Apollo SciTech Model AS-ALK2	1
Northeastern University Marine Science Center	VINDTA 3C (Marine Analytics and Data)	1
Massachusetts Institute of Technology	Custom built by Andrew Dickson Laboratory UCSD	1
Woods Hole Oceanographic Institution	Metrohm 808 Titrando	1
University of Connecticut	Contros HydroFIA	2
University of New Hampshire	Contros HydroFIA	2

**Table 2.** Number of sampling stations, mean total alkalinity (TA), and salinity by subregion. Values in parentheses are standard deviation. The number of observations may differ between TA and salinity due to quality control measures for each parameter.

Region	Number of stations	TA (*μ*mol kg^−1^)	Number of observations	Salinity	Number of observations

GOM	22	1899.9 (335.8)	60	26.1 (7.1)	60
CCB	33	1813.0 (457.7)	95	25.1 (8.5)	99
BB/VS	16	1824.8 (253.8)	48	27.0 (5.5)	43
NB	6	1968.3 (37.1)	15	31.9 (1.7)	14
LIS	9	1881.3 (378.7)	9	20.4 (9.8)	23

**Table 3.** Summary fit statistics including number of samples (*N*), *r*^2^, p, and root mean square error (RMSE) for multiple linear regression analysis predicting total alkalinity from temperature and salinity for all data combined (All Data) and individual subregions of the northern Gulf of Maine (GOM), Cape Cod Bay/Central Gulf of Maine (CCB), Buzzards Bay/Vineyard Sound (BB/VS), Narragansett Bay (NB) and Long Island Sound (LIS). Fits for all data also include latitude as a predictor variable. Full fit statistics can be found in table S1.

Region	*N*	*r* ^2^	*p*	RMSE (*μ*mol kg^−1^)

All Data	191	0.894	<0.0001	121.1
GOM	45	0.773	<0.0001	177.1
CCB	84	0.944	<0.0001	110.9
BB/VS	34	0.814	<0.0001	70.3
NB	14	0.176	0.566	33.5
LIS	14	0.674	0.0085	48.7
