# Supplementary material for: Synoptic assessment of coastal total alkalinity through community science
Source: Environ Res Lett. Author manuscript; Available in PMC 2022 Jan 21. (PMC8780830; doi:10.1088/1748-9326/abcb39)
Supplement: Sup 1 [file NIHMS1717915-supplement-Sup_1.pdf]

Supplement to Rheuban et al. “Synoptic Assessment of Coastal Total Alkalinity through Community Science"

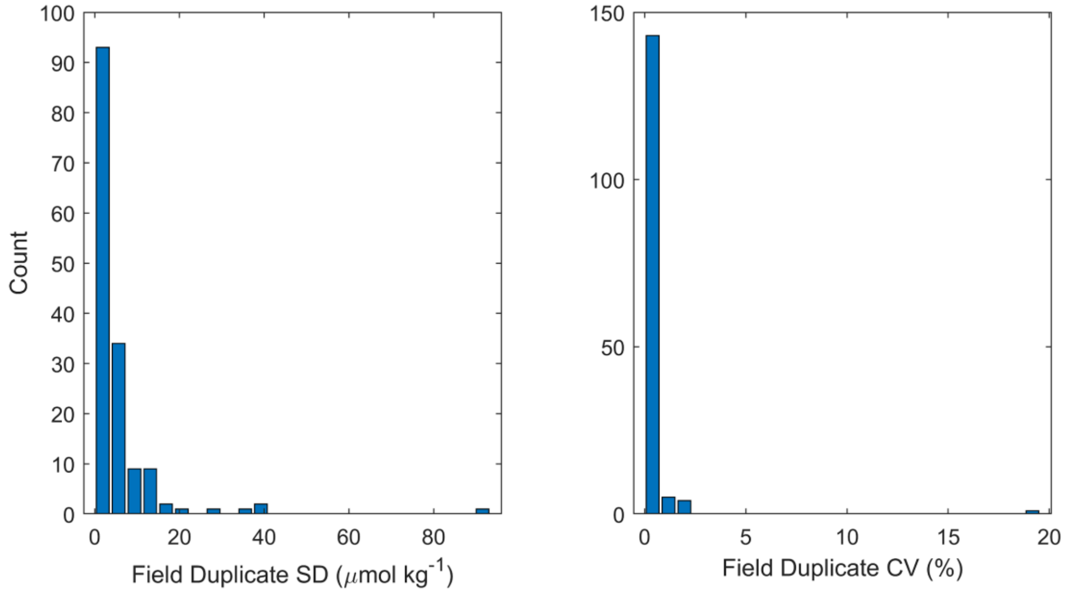

Figure S1. Histogram of standard deviation (left) and coefficient of variation (right) between field duplicates.

Although this manuscript describes correlations consistent across the Northeast region and various subregions, there are also likely distinct salinity-alkalinity relationships at the watershed spatial scale. This dataset is extensive spatially, but it is limited at the individual watershed/embayment scale, with most embayments having only a single sampling station and thus at most 3 distinct datapoints, which is not enough data to evaluate a salinity-alkalinity relationship for a single station. Figure S2 illustrates the myriad of different salinity-alkalinity relationships that may exist within the Shell Day dataset by illustrating each sampling station as data points connected by lines.

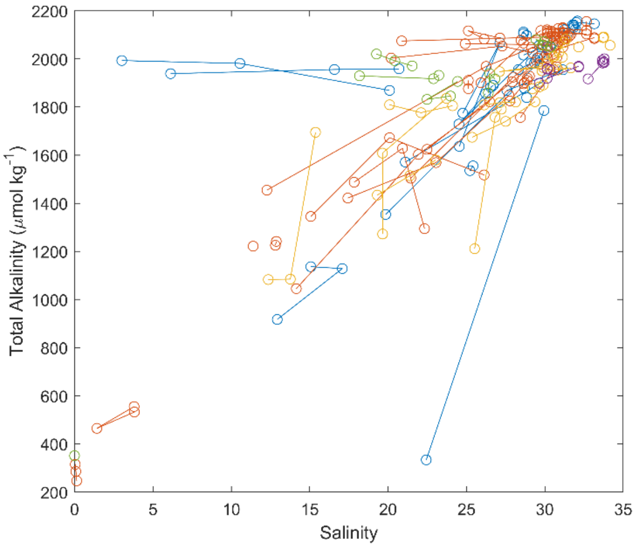

Figure S2. Total alkalinity vs. salinity for all Shell Day data, analogous to Figure 3. In this graphic, data from individual stations are connected by lines and colored by region, which match the map shown in Figure 1

Table S1. Coefficients for simple linear regression analysis predicting total alkalinity (TA) from salinity for the five subregions: Gulf of Maine (GOM), Cape Cod Bay (CCB), Buzzards Bay/Vineyard Sound (BB/VS), Narragansett Bay (NB) and Long Island Sound (LIS), and all data combined (All Data). Numbers in parentheses indicate the standard error for each fit parameter. GOM_{lab} indicates fits from the GOM region using laboratory salinometer measurements. Bold coefficients indicate statistical significance at the $p < 0.01$ level.

Region	N	RMSE	r^2	Intercept	Salinity
GOM	48	188.6	**0.733**	-93.6 (178.3)	**70.3 (6.3)**
CCB	84	113.7	**0.941**	**422.3 (40.1)**	**54.0 (1.5)**
BB/VS	34	75.4	**0.787**	**828.6 (99.4)**	**37.3 (3.4)**
NB	14	34.4	0.133	**1705.4 (193.5)**	8.2 (6.1)
LIS	14	75.2	0.221	**1692.7 (152.3)**	10.8 (5.8)
GOM_{lab}	10	30.4	**0.993**	**103.3 (53.3)**	**64.1 (1.9)**
All Data	194	157.3	**0.820**	**472.0 (48.5)**	**51.1 (1.7)**

Table S2. Coefficients for multiple linear regression analysis predicting total alkalinity (TA) from temperature, salinity, and latitude for the five subregions: Gulf of Maine (GOM), Cape Cod Bay (CCB), Buzzards Bay/Vineyard Sound (BB/VS), Narragansett Bay (NB) and Long Island Sound (LIS). GOM_{lab} indicates fits from the GOM region using laboratory salinometer measurements, and All Data indicates the combined fit for the entire region (Eq. 1). Fits were of the form from Alin et al. 2012, and included interactions terms. Numbers in parentheses indicate the standard error for each fit parameter. Bold coefficients indicate statistical significance at the $p < 0.01$ level, and italics indicate significance at the $p < 0.05$ level.

Region	N	RMSE	R^2	Intercept	Salinity	Temp (°C)	S*T
GOM	45	177.1	**0.773**	**1876.4 (29.8)**	**62.3 (6.7)**	*-30.8 (12.7)*	*6.7 (2.9)*
CCB	84	110.9	**0.944**	**1794.1 (13.6)**	**53.5 (1.8)**	-8.2 (4.7)	-0.3 (0.8)
BB/VS	34	70.3	**0.814**	**1883.8 (21.8)**	**38.1 (5.4)**	-3.1 (8.2)	-3.1 (2.6)
NB	14	33.5	0.176	1958.9 (15.7)	7.1 (8.2)	2.0 (11.0)	-6.7 (9.2)
LIS	14	48.7	**0.674**	1933.0 (28.0)	-7.1 (8.3)	*-37.5 (14.2)*	-6.6 (3.3)
GOM_{lab}	10	26.8	**0.995**	**1929.3 (28.7)**	**53.2 (9.6)**	-13.9 (14.4)	3.2 (2.6)

Region	N	RMSE	R^2	Intercept	Salinity	Temp (°C)	S*T	Lat	S*Lat	T*Lat
All Data	191	121.1	**0.894**	**4958.9 (510.8)**	**-571.7 (73.9)**	-29.2 (120.1)	0.8 (0.7)	**-72.3 (12.0)**	**14.4 (1.7)**	0.2 (2.8)

Our expectation was that the lower precision and accuracy of refractometers would be responsible for the anomalous relationships between the variability in salinity and the variability in total alkalinity. However, higher precision multiparameter datasondes were used at most of the sampling stations that displayed the anomalous variability and salinity and alkalinity (e.g. Groups 2 and 3, Figures 4 and S3). This implied that the observed patterns in variability was not driven by low precision or accuracy of instrumentation.

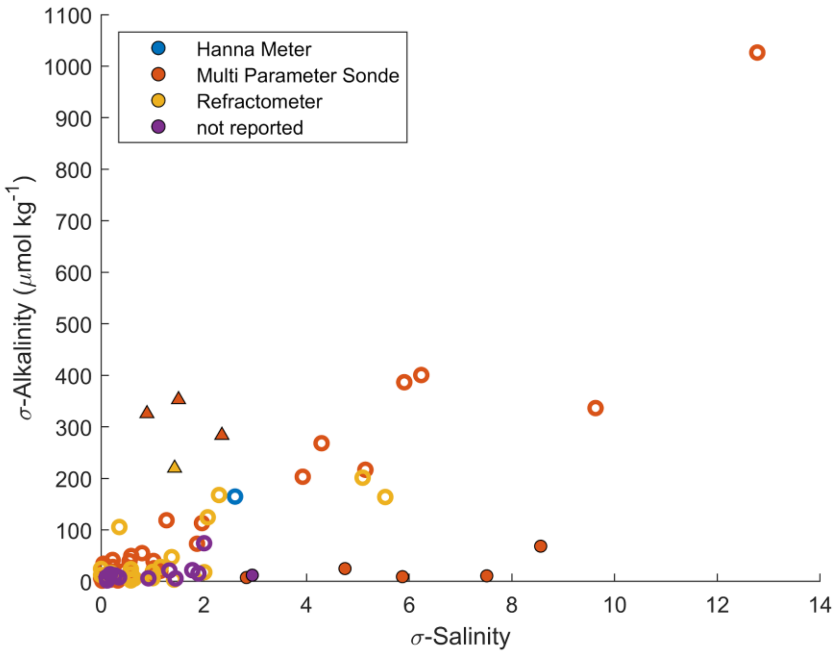

Figure S3. Standard deviation in alkalinity vs standard deviation in salinity over a tidal cycle from each sampling station (analogous to Figure 4). Symbols and filled or open data points follow the observed tidal variation regimes illustrated in Figure 4. Data points are colored by method of salinity collection.

To provide context for our total alkalinity samples, and to identify potential contributions of high-alkalinity freshwater at stations sampled during Shell Day, we evaluated TA measurements from the USGS Water Data for the Nation database (https://nwis.waterdata.usgs.gov/nwis). Water quality field/lab data were searched using the parameter codes 00418, 29801, 29802, 29803, 39086, and 39087, which reflect multiple methods of alkalinity determination (Table S2), and 14808 measurements were found. Measurements from each station were averaged across the entire dataset and mapped for context (Figure S4).

Table S2. Parameter codes and descriptions from the USGS Water Data for the Nation database.

Parameter Code	Description
00418	Alkalinity, water, filtered, fixed endpoint (pH 4.5) titration, field, milligrams per liter as calcium carbonate
29801	Alkalinity, water, filtered, fixed endpoint (pH 4.5) titration, laboratory, milligrams per liter as calcium carbonate
29802	Alkalinity, water, filtered, Gran titration, field, milligrams per liter as calcium carbonate
29803	Alkalinity, water, filtered, Gran titration, laboratory, milligrams per liter as calcium carbonate
39086	Alkalinity, water, filtered, inflection-point titration method (incremental titration method), field, milligrams per liter as calcium carbonate
39087	Alkalinity, water, filtered, inflection-point titration method (incremental titration method), laboratory, milligrams per liter as calcium carbonate

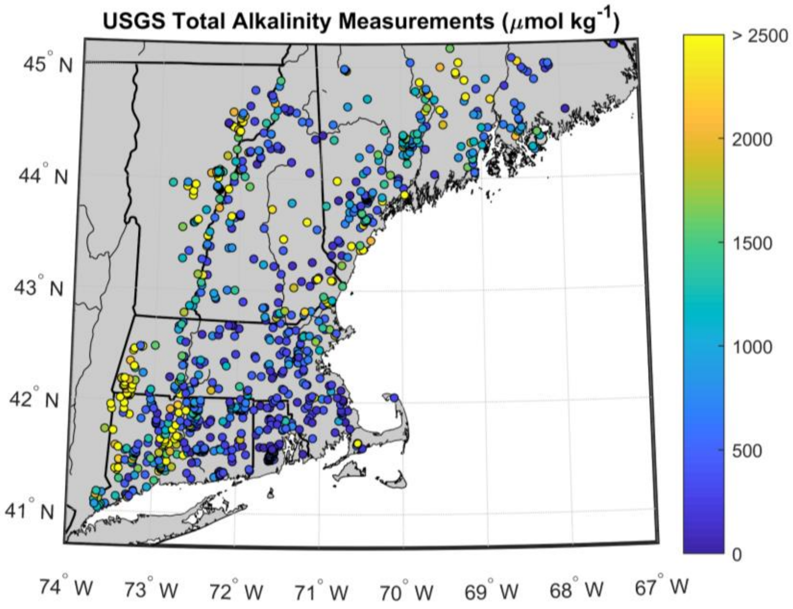

Figure S3. Mean alkalinity reported in the USGS Water Quality Samples for the Nation database for the New England region. Major rivers are indicated on the map. The color scale has been maximized at 2500 umol kg^{-1} for relevance to typical seawater values. Note that the maximum average value per station was 10451 umol kg^{-1}.

.

List of Participating Water Monitoring Organizations

1 Aquacultural Research Corp, 2 Barnstable Clean Water Coalition, 3 Boothbay Region Land Trust, 4 Boston Harbor Island National and State Park, 5 Belfast Bay Watershed Coalition, 6 Bigelow Laboratory, 7 Buzzards Bay Coalition, 8 Cape Cod Cooperative Extension, 9 Committee for the Great Salt Pond, 10 Connecticut Fund for the Environment (Save the Sound), 11 Clean Up Sound and Harbors, 12 Casco Bay Estuary Partnership, 13 Center for Coastal Studies, 14 Cohasset Center for Student Coastal Research, 15 Derecktor Shipyards, 16 Downeast Institute, 17 EPA Atlantic Coastal Environmental Sciences Division, 18 EPA Region 1, 19 Earthwatch/Schoodic Institute, 20 Friends of Casco Bay, 21 Falmouth Water Stewards/Pond Watch, 22 Harbor Watch, 23 Hurricane Island Center for Science and Leadership, 24 Interstate Environmental Commission, 25 Island Creek Oysters, 26 Island Institute, 27 Kennebec Estuary Land Trust, 28 Maine Coastal Observers Alliance, 29 Maine Department of Environmental Protection, 30 Maine Maritime Academy, 31 Marine Biological Lab, 32 Martha's Vineyard Commission, 33 Martha's Vineyard Shellfish Group, 34 Massachusetts Maritime Academy, 35 Mook Sea Farm, 36 North and South River Watershed Association, 37 Nantucket Land Bank, 38 Nantucket Land Council, 39 Nantucket Natural Resources Department, 40 Neponset River Watershed Association, 41 New England Aquarium, 42 New England Science and Sailing, 43 Pleasant Bay Alliance (Friends of Pleasant Bay), 40 Rockport Conservation Commission, 41 Water for ME Foundation, 44 Salem Sound Coastal Watch, 45 Swampscott Conservancy, 46 Salt Ponds Coalition, 47 Save Bristol Harbor, 48

Save the Bay, 49 Setauket Harbor Task Force, 50 Shaw Institute, 51 Town of Mashpee Department of Natural Resources, 52 University of Maine-Machias, 53 University of New Hampshire, 54 UNH Marine Docents, 55 URI Watershed Watch, 56 University of New England, 57 Wampanoag Tribe of Gay Head (Aquinnah), 58 Woods Hole Oceanographic Institution, 59 Woods Hole Sea Grant.
